# Supplementary material for: Heavy footprints of upper-ocean eddies on weakened Arctic sea ice in marginal ice zones
Source: Nat Commun. 2022 Apr 20;13:2147. doi: 10.1038/s41467-022-29663-0 (PMC9021234; doi:10.1038/s41467-022-29663-0)
Supplement: Supplementary file 1 — Supplementary Information [file 41467_2022_29663_MOESM1_ESM.pdf]

Supplementary Information

Heavy footprints of upper-ocean eddies on weakened Arctic sea ice in marginal ice zones

G.E. Manucharyan[1] and A.F. Thompson[2]

[1]*University of Washington, School of Oceanography, Seattle, Washtingon, USA*
[2]*California Institute of Technology, Environmental Science and Engineering, Pasadena, California, USA*

1 Marginal ice zones from space

Signatures of ocean eddies at the sea ice margins are evident from satellite imagery as a convoluted ice edge structure with length scales comparable to the oceanic deformation radii (Fig. 1). An example of satellite-derived sea ice concentrations (Synthetic Aperture Radar snapshot) in the Fram Strait (Fig. 1, left) shows structures consistent with oceanic eddy variability exhibited in fronts, filaments, vortices, and striations. The characteristic eddy-like patterns, spatial scales of 10-50 km, and persistence times of several days point to coupled dynamics between sea ice and ocean eddies. A similar example is presented from the East Siberian Shelf away from any strong boundary currents (Fig. 1, right), where satellite imagery suggests that sea ice can be localized or trapped in small-scale eddies, filaments, fronts, and wave-like patterns. Such images could be commonly found in marginal ice zones if winds are slow, particularly when sea ice is low-concentrated and growing or melting.

2 Supplementary Movie 1: Time-evolution of sea ice and vorticity

The video demonstrates the evolution of sea ice and upper ocean vorticity starting from August 2011 and progressing through the strong melting year ending in September 2012, as simulated by the high-resolution ocean-sea ice model LLC4320. The left panel shows sea ice concentration (color) and volume per unit area (purple, green, and yellow contour lines denote 1,2,3 meters). The right panel shows the ocean vorticity at 20 meters depth normalized by the Coriolis parameter f. The ice-free ocean is masked in black, and the land is masked in white. Note the sea ice transition from the viscous-plastic fluid regime that is accompanied by linear kinematic features towards a nearly passive tracer regime within and near marginal ice zones, particularly evident at the end of summer. This passive tracer regime spreads to occupy a significant portion of the Arctic sea ice. The high-resolution LLC4320 model is not a reanalysis-type model and hence was not adjusted to accurately reproduce observations. As a result, there is an apparent mismatch in the locations of summer sea ice retreat: compared to the observed 2012 sea ice state, the model overestimates the sea ice cover in the Western Arctic ocean and underestimates its melt in the Eastern Arctic. Nonetheless, the high-resolution simulation provides a unique opportunity to explore the critical role of small-scale MIZ dynamics. These fundamental dynamical processes should be ubiquitous to the MIZ regardless of exact geographical location and size.

3 Mechanical sea ice response to eddies: theoretical considerations

Next, we consider ocean and ice evolution equations to arrive at theoretical arguments that support some of the main conclusions of our study. First, consider a frontal filament in the ocean, a feature typically associated with the edges of coherent mesocale eddies. In the along-filament y-direction, ocean currents are in near-geostrophic balance (i.e.,

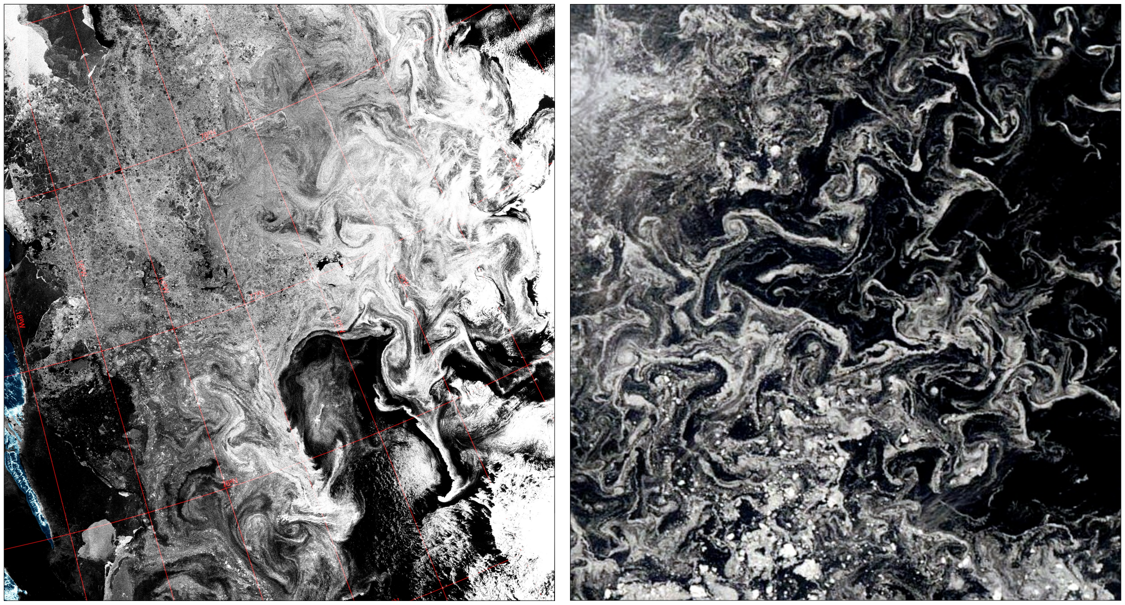

Figure S1: Examples of satellite MIZ images displaying signatures of small-scale ocean eddies, fronts, and filaments in Fram Strait (Synthetic Aperture Radar snapshot, resolution of 40 m) shown in the left panel and over the East Siberian Shelf in the Arctic Ocean (Terra/MODIS reflectance snapshot, resolution of 0.5 km) shown in the right panel. Characteristic eddy length scales are about 10-30 km, whereas fronts and filaments are O(5 km) thick and O(100 km) long. Images are taken during cloud-free days with relatively low winds which allowed the sea ice distribution time to adjust to upper-ocean turbulence patterns.

$fv_o \approx \phi_x$), where ϕ is the sea surface height potential multiplied by gravitational acceleration. In the cross-filament x-direction, there is a weaker convergent ageostrophic flow that plays a critical role in driving sea ice accumulation. Over the idealized filaments and eddies, it is possible to solve for sea ice velocity and concentrations by simplifying the influence of rheology down to ice viscous stresses in the x-direction and compressional force in the y-direction. We consider the continuous formulation of sea ice equations of motions:

$$\frac{\partial \mathbf{u}_i}{\partial t} = -f\mathbf{k}\times\mathbf{u}_i + c\boldsymbol{\tau}_{io}/m_i - \nabla\phi + \nabla\cdot\boldsymbol{\sigma}/m_i, \tag{1}$$

$$\frac{\partial \mathbf{u}_o}{\partial t} = -f\mathbf{k}\times\mathbf{u}_o - c\boldsymbol{\tau}_{io}/m_o - \nabla\phi, \tag{2}$$

$$\frac{\partial c}{\partial t} + \nabla(\mathbf{u}_i c) = 0, \tag{3}$$

$$\tau_{io} = \rho_o C_d(\mathbf{u_o} - \mathbf{u}_i)|\mathbf{u}_o - \mathbf{u}_i|, \tag{4}$$

where $\mathbf{u}_i$ is the horizontal sea ice velocity, $m_i = \rho_i c\, h$ is the mass of ice per unit area, also called the effective thickness, $m_o = \rho_o h_m$ is the mass of the ocean's surface mixed layer (with depth h_m) per unit area, ρ_i is the density of ice, c is concentration, h is characteristic thickness, f is the Coriolis parameter, σ is the stress tensor representing sea ice rheology, τ_{io} is ice-ocean stress associated with differences between the ice $\mathbf{u}_i$ and surface ocean speed $\mathbf{u}_o$, and C_d is the drag coefficient. The non-linear sea ice momentum advection represents a minor contribution in Eq. 1 and is typically neglected in numerical models. We also neglect the non-linear term in the ocean's momentum equation under the assumption of small Rossby number. Finally, in what follows, we assume h is constant because thermodynamically, the ice thickness evolves on timescales that are longer than the those associated with the evolution of short-lived (O days) ocean filaments. Furthermore, in relatively low-concentrated areas, the sea ice divergence/convergence leads primarily to changes in concentration rather than in average thickness.

The limit of sea ice acting as a passive tracer (i.e. $\mathbf{u}_i = \mathbf{u}_o$) is achieved when the rheology term is negligible and the sea ice thickness m_i is negligibly small. Note that even in the case where the rheological term in (1) is identically

zero, sea ice inertia will produce slight differences between the ice and ocean velocities and an associated ice-ocean stress that can be balanced by the Coriolis force, i.e. the classical Ekman balance. In this limit upper-ocean cyclones, or low pressure systems, will have a convergent Ekman transport and lead to sea ice accumulation, and anticyclones will have a divergent Ekman transport and repel the ice. However, as sea ice concentrations rise, the rheology terms also become important and counteract the ice accumulation by eddies.

The rheology of seawater, i.e. turbulent Reynolds stresses, is negligible compared to the rheology of sea ice as the effective turbulent ocean viscosity is much smaller than sea ice viscosity. Additionally, sea ice inertia is much smaller than that of the ocean, i.e. $m_i << m_o$, because ice thickness is much thinner than the mixed layer depth. As a result, a simplified equation describing the difference between ice and ocean velocities when the ocean is in near-geostrophic balance (*i.e.* low Rossby number which are indeed observed in our simulation) is given by:

$$f\mathbf{k}\times(\mathbf{u_i}-\mathbf{u_o})+cm_i^{-1}\rho C_d(\mathbf{u_i}-\mathbf{u_o})|\mathbf{u_i}-\mathbf{u_o}| = m_i^{-1}\nabla\cdot\boldsymbol{\sigma}. \tag{5}$$

From this relationship, $u_i = u_o$ can only occur if the rheology term is negligible, *e.g.* when sea ice acts as a passive tracer and ice floes are sufficiently small so they do not interact with each other. In steady state, when the ice mass has been redistributed, the equation is even simpler for a filament since $u_i = u_o = 0$, and only $v_i, v_o \neq 0$ (similar could be written for a symmetric eddy in radial coordinates). The y and x components of (5) become, respectively:

$$cm_i^{-1}\rho_o C_d(v_i - v_o)|v_i - v_o| = m_i^{-1}[\nabla\cdot\boldsymbol{\sigma}]^2, \tag{6}$$

$$f(v_i - v_o) = m_i^{-1}[\nabla\cdot\boldsymbol{\sigma}]^1, \tag{7}$$

where superscripts $1,2$ correspond to the components of the rheology force $\nabla\cdot\boldsymbol{\sigma}$ in the x and y directions.

The viscous-plastic rheology formulation defines σ as:

$$\sigma_{i,j} = 2\eta\dot{\varepsilon}_{i,j} - (\zeta-\eta)\dot{\varepsilon}_{k,k}\delta_{i,j} - 0.5P\delta_{i,j}, \tag{8}$$

where η and ζ are bulk and shear viscosities, respectively, which are functions of the sea ice strain rate $\dot{\varepsilon}$

$$\dot{\varepsilon}_{i,j} = \frac{1}{2}\left(\frac{\partial u_i}{\partial x_j} + \frac{\partial u_j}{\partial x_i}\right), \tag{9}$$

and maximum sea ice pressure $P = P^* ch \exp[-C^*(1-c)]$. The indices i, j, k have values $1,2$, denoting the two-dimensional coordinate system used in the sea ice model. The viscosities depend on a strain tensor invariant Δ in such a way that the principle components of the stress lie on an elliptical curve with a ratio of $e = 2$ of major to minor axis:

$$\zeta = min\left(\frac{P}{2max(\Delta,\Delta_{min})}, \zeta_{max}\right) \text{ and } \eta = \zeta e^{-2}, \tag{10}$$

$$\Delta = \left[(\dot{\varepsilon}_{11}^2 + \dot{\varepsilon}_{22}^2)(1+e^{-2}) + 4e^{-2}\dot{\varepsilon}_{12}^2 + 2\dot{\varepsilon}_{11}\dot{\varepsilon}_{22}^2(1-e^{-2})\right]^{0.5}. \tag{11}$$

For a complete description of the viscous-plastic rheology see [1, 2].

In order to simplify the equations shown above and to gain critical insights into the dynamics, we consider an idealized case of sea ice behavior over geostrophic flow in the y-direction and assume homogeneity along the y direction, *i.e.* all $\partial/\partial y \equiv 0$. In steady state, mass conservation requires that $u_i = 0$ and only $v_i \neq 0$. In this case the strain rate tensor reflects a pure shear $\dot{\varepsilon}_{11} = \dot{\varepsilon}_{22} = 0$, $\dot{\varepsilon}_{12} = 0.5\partial_x v_i$, and $\Delta = |\dot{\varepsilon}_{12}|$. In this simplified state, the stress tensor only contains the shear viscosity and pressure terms:

$$\sigma_{ij} = 2\eta\dot{\varepsilon}_{ij} - 0.5P\delta_{ij}. \tag{12}$$

Calculating the divergence of the stress tensor, there are forces in the x-direction: $[\nabla\cdot\sigma]_1 = -0.5\partial_x P$ and in the y-direction $[\nabla\cdot\sigma]_2 = \partial_x(\eta\partial_x v_i)$. Hence the steady-state equations governing the distribution of sea ice velocity and concentration become:

$$c\rho_o C_d(v_i - v_o)|v_i - v_o| = \partial_x(\eta\partial_x v_i) \tag{13}$$

$$\rho_i chf(v_i - v_o) = -0.5\partial_x P. \tag{14}$$

While, Eqns. 13, 14 provide a simplified representation of ice-ocean interactions, they are sufficient to highlight key qualitative dynamics. Eq. 13 shows that ice and ocean velocities can not be equal, otherwise the viscous term

$\partial_x(\eta \partial_x v_i)$ would introduce a non-zero force due to strongly shearing ice currents; note that ocean currents are allowed to be strongly sheared because of the relatively small turbulent stresses in the ocean as compared to internal sea ice stresses. If $v_i - v_o \neq 0$ then, based on Eq. 14, $\partial_x P \neq 0$ and hence $\partial_x c \neq 0$. This implies that there will be a direct response of the sea ice concentration field to the drag from underlying ocean eddies. An important limit is when the right-hand-side rheology terms in Eqns. 13, 14 are of the same order – a limit that occurs when the maximum and minimum bounds viscosity and strain rates in Eqn. 10 are not realized. The limit where the shear and normal internal sea ice stresses are of the same order of magnitude ($\eta |\partial_x v_i| \sim 0.5P$) is due to the VP rheology that specifies the yield curve to be an ellipse with a relatively low eccentricity of 2 [1]. In this limit, a scaling law arises for the velocity (or vorticity) difference between the ocean and the sea ice

$$v_i - v_o \sim \frac{fh}{C_d} \text{ or } \frac{\zeta_i - \zeta_o}{f} \sim \frac{h}{C_d L}. \tag{15}$$

Here, we have assumed that $\rho_i/\rho_o = O(1)$ and L is associated with the eddy scale. The scaling law holds only when the ice is sufficiently weak to permit strong strain rate. Contrary, when the ice is very strong (at high concentrations and thicknesses), the rheological forces would balance all other forces even with very weak of negligible sea ice strain rate, such that one could assume $v_i \approx 0$ in Eqns. 13 and 14.

The scaling laws breaks down if strong large-scale atmospheric winds translate free-drifting sea ice relative to ocean eddies so fast that it cannot pick up vorticity from ocean eddies. The intrinsic adjustment timescale characterizing the momentum exchange between the ice and the ocean can be estimated by considering a simplified problem of how a free-drifting ice with an initial ice-ocean velocity difference $\Delta v_0 > 0$ equilibrates subject to no other forcing except for the ice-ocean drag. Writing a balance between the sea ice momentum tendency and the ice-ocean stress (and ignoring the Coriolis and sea ice rheology) we arrive at:

$$h\rho_i \Delta \dot{v} = -\rho_o C_d \Delta v^2, \tag{16}$$

$$\frac{\Delta v}{\Delta v_0} = \frac{1}{1 + (\rho_o/\rho_i) C_d (\Delta v_0/h) t}. \tag{17}$$

The solution above implies that the relative ice-ocean velocity approaches zero as $(t/T)^{-1}$ with a characteristic timescale $T \approx h\ (C_d\ \Delta v_0)^{-1}$, where $\rho_o/\rho_i \approx 1$ was used. Taking as characteristic values $h = 1$ m, $C_d = 3 \times 10^{-3}$, and $\Delta v_0 = 0.1$ m s^{-1} gives an equilibration timescale T of about an hour. This also implies that the relaxation of freely-drifting sea ice vorticity towards the underlying ocean vorticity occurs over the same timescale, assuming the vorticity input from the atmospheric wind stress is negligible compared to the vorticity of the ice-ocean stress. This assumption is expected to be valid because the atmosphere-ice and ocean-ice stresses are of the same order of magnitude while the atmospheric eddy length scales are at least an order of magnitude larger than oceanic.

Thus, the scaling laws suggest that when the sea ice is mobile, the ice-ocean stress in the momentum and vorticity budgets places strong constraints on the difference between the upper-ocean and the sea-ice velocity (when winds are low) and vorticity (when length scales of winds is much larger compared to ocean currents). This relationship also impacts ocean energetics, as the upper-ocean eddy dissipation is substantially weakened when the sea ice and ocean vorticities are correlated: this regime occurs predominantly in summer-time marginal ice zones (see Figure 4 of main text).

4 Mechanical sea ice response to eddies: idealized simulations

Here we present the sensitivity study of sea ice dynamics in response to ocean eddies and subject to different rheology parameters. Numerical sea ice simulations were performed using the MITGCM with the viscous-plastic rheology based on Hibler 1979. To mimic the eddying marginal ice zone dynamics as was observed from satellites (see Figure 1 in the main text) and simulated in the high-resolution ocean-ice model (Figure 2), we explore the sea ice behavior over an idealized grid of prescribed cyclonic and anticyclonic eddies (Figure S2, a). We initialized the sea ice with a state of rest and a large-scale concentration distribution ranging linearly from zero (open ocean) to 100% cover along the x-axis, with a uniform concentration along y-axis. The sea ice is free to evolve subject to sea surface height and ice-ocean drag forcing due to ocean currents, which are stationary in time. We then explore the sensitivity to the critical rheology parameter, C^*, which affects the dynamical transitions in sea ice behavior occurring at a particular sea ice concentration c. In viscous-plastic formulation (Hibler 1979) the bulk and shear sea ice viscosity, as well as the response of the ice to

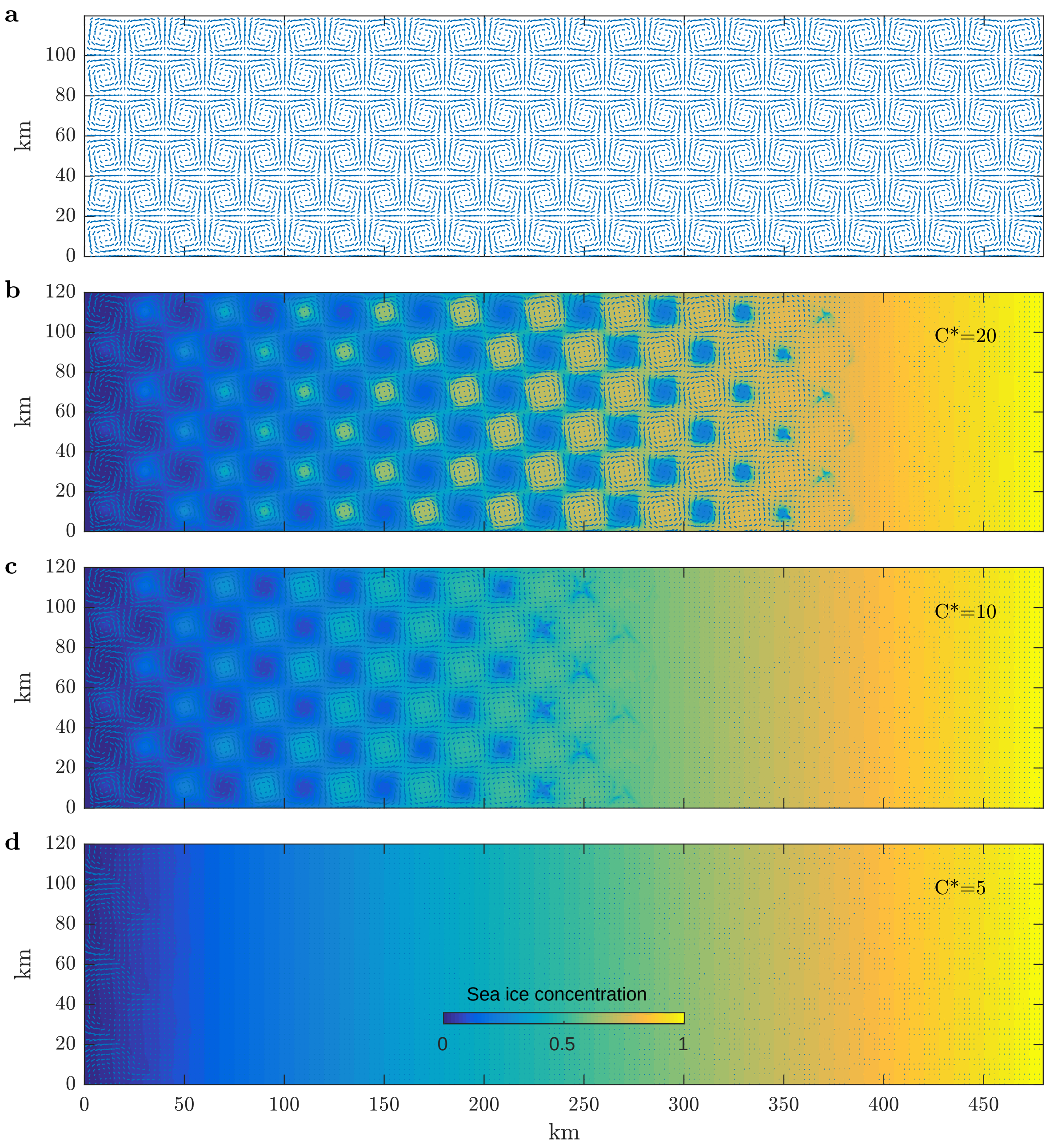

Figure S2: Sea ice response to an underlying eddying ocean for various values of rheology parameter C^*. **a.** Idealized non-divergent ocean flow field defined by a streamfunction $\psi \sim sin(2\pi x/L) * sin(2\pi y/L)$ with a wavelength $L = 40$ km. **b,c,d**. Sea ice concentration (colors) and velocity (arrows with maximum length corresponding to about 0.1 m/s speed) for the rheology parameters $C* = 20, 10, 5$, respectively, achieved 12 hours after being subject to the eddying ocean flow in **a**. There is a relatively sharp transition in mean concentrations from regions of relatively immobile sea ice to one that strongly reflects the underlying ocean eddies. Note the redistribution of sea ice concentration, being concentrated over cyclones and reduced over anticyclones.

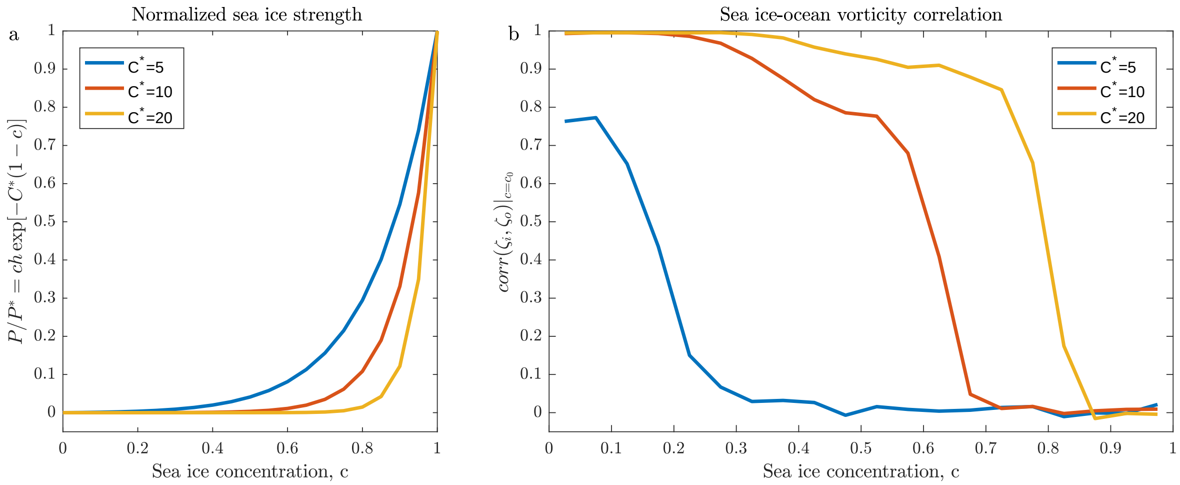

Figure S3: (**a**) Sea ice strength parameter, P, based on Hibler 1979 rheology and (**b**) correlation between the upper-ocean and sea ice vorticities arising in numerical experiments shown in Figure S2 plotted for different sea ice concentration bins. Three different curves in each panels represent sensitivities to the rheology parameter $C^* = 5, 10, 20$, as in Figure S2.

convergent flow, are proportional to its strength $P \sim c\exp[-C^*(1-c)]$ (Figure S3, a). Current GCMs that implement this formulation use an empirically-determined value of $C^* = 20$, based on Arctic-wide observations. Hence, this particular value of C^* is not necessarily representative of marginal ice zones, or localized regions with varying types of sea ice. Here we test the sea ice response to various values of $C^* = 5, 10, 20$, and demonstrate that C^* governs the existence of a critical sea ice concentration c_{cr} below which the sea ice velocity and concentration strongly reflect the underlying ocean eddies with ice accummulating in cyclones and being repelled from anticyclones. Above the critical concentration, the sea ice is largely insensitive to the underlying ocean eddy forcing (Figure S2). The concentration becomes critical, c_{cr}, when the magnitude of the rheological force becomes comparable to ice-ocean stress due to eddies, i.e. $\nabla P(c_{cr}) \sim \tau$. Below the critical concentration, the sea ice is approximately in free drift and the scaling laws dictate a close link between the ice and the ocean vorticity.

Recall that the time scale associated with the sea ice response to ocean eddies is about an hour, which is short but not negligible. On the other hand, for the sea ice concentration patterns to emerge in response to ocean eddies, an advective time scale is most relevant and is of the order of several hours to a day, depending on the strength of the underlying eddy field. Therefore, we only expect the sea ice concentration patterns to reflect ocean eddies if the atmospheric forcing over this period is sufficiently weak that it does not decouple the ice and the eddy. Under the influence of strong winds, eddy patterns are less likely to to be evident but the sea ice vorticity is still expected to be correlated with the ocean vorticity, as long as the length scale of winds is much larger than ocean eddies.

5 Evolution of ocean characteristics in different regions

We split the Arctic Ocean domain of the LLC4320 model into non-overlapping 500 by 500 km sub-domains. We kept for analysis only those sub-domains that contain non-zero year-round sea ice concentration, resulting in 18 sub-domains. For each sub-domain we take spatial averages of physical ocean characteristics and perform a 30 day running mean on the time series, keeping only points separated by 15 days.

References

[1] WD Hibler III. A dynamic thermodynamic sea ice model. *Journal of Physical Oceanography*, 9(4):815–846, 1979.

[2] Jinlun Zhang and WD Hibler. On an efficient numerical method for modeling sea ice dynamics. *Journal of Geophysical Research: Oceans*, 102(C4):8691–8702, 1997.

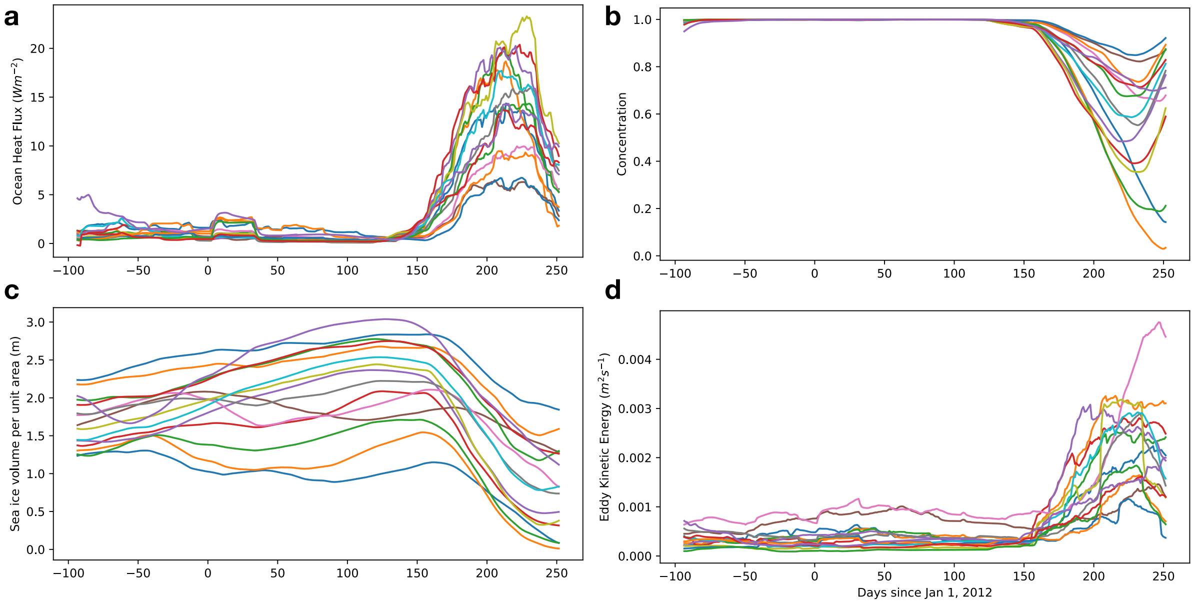

Figure S4: Seasonal evolution of key ocean and sea ice characteristics in 500x500 km sub-domains in the Arctic Ocean. Time series of **(a)** ocean-ice heat flux (W m^{-2}), **(b)** sea ice concentration, **(c)** sea ice volume per unit area (m), and **(d)** eddy kinetic energy (m^2 s^{-2}) plotted for the 18 of the sub-domains described in Section S5; colors represent different sub-domains, consistently between all panels. These time series are used to determine the strength of the EKE-heat flux- concentration feedback.

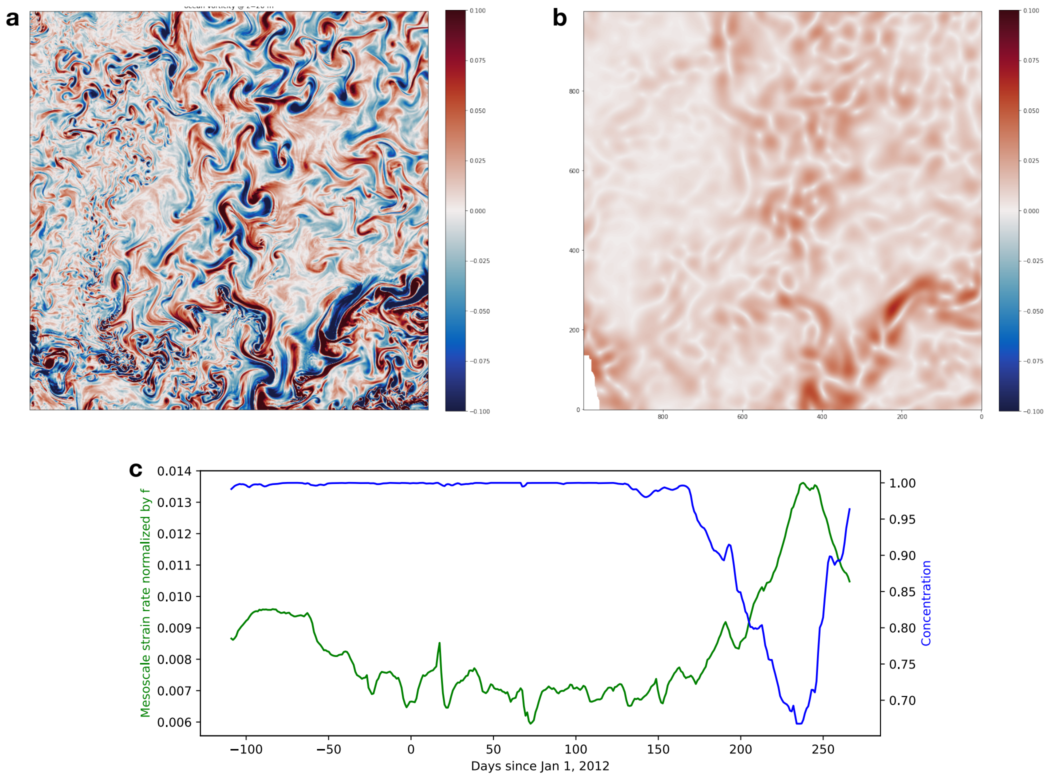

Figure S5: Examples of the eddy field and the associated strain variability. (**a**) Ocean vorticity at 20 m depth normalized by the Coriolis parameter f plotted for the 500×500 km sub-domain shown as a green box in Figure 1c of the main text. (**b**) The magnitude of the oceanic mesoscale strain for the same sub-domain, calculated from the velocity field smoothed by a Gaussian convolutional filter with a radius of 30 km. (**c**) The seasonal evolution of the subdomain-averaged sea ice concentration and the magnitude of the mesoscale strain rate normalized by f.

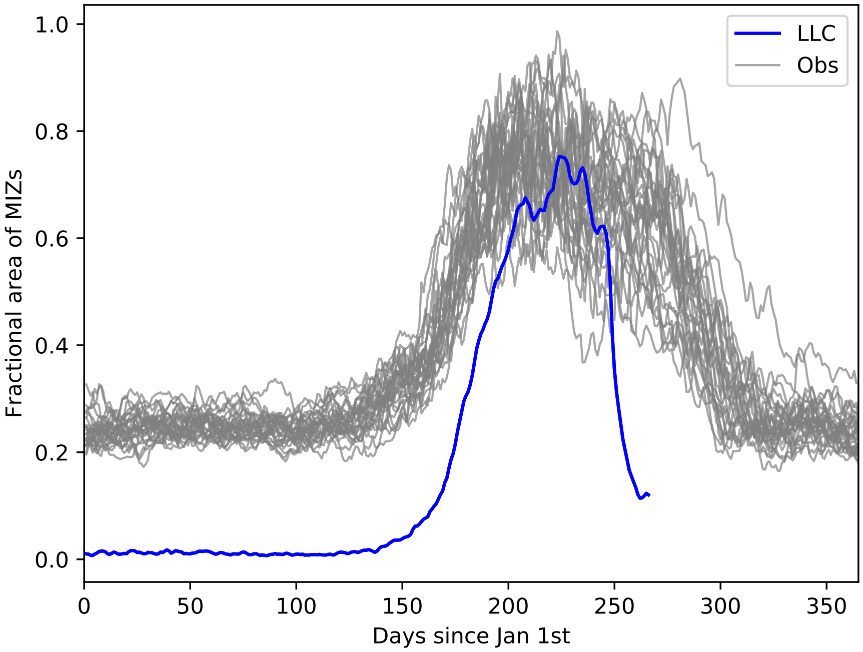

Figure S6: The percentage of sea ice area occupied by the marginal ice zone. Gray curves show observations for different years (NSIDC), while the black curve shows the LLC4320 model simulation.
