# Supplementary material for: Heavy footprints of upper-ocean eddies on weakened Arctic sea ice in marginal ice zones
Source: Nat Commun. 2022 Apr 20;13:2147. doi: 10.1038/s41467-022-29663-0 (PMC9021234; doi:10.1038/s41467-022-29663-0)
Supplement: Supplementary file 3 — Description of Additional Supplementary Files [file 41467_2022_29663_MOESM3_ESM.pdf]

Description of Additional Supplementary Files

File Name: Supplementary Movie 1
Description: **Time-evolution of sea ice and vorticity.**

The video demonstrates the evolution of sea ice and upper ocean vorticity starting from August 2011 and progressing through the strong melting year ending in September 2012, as simulated by the high-resolution oceansea ice model LLC4320. The left panel shows sea ice concentration (color) and volume per unit area (purple, green, and yellow contour lines denote 1,2,3 meters). The right panel shows the ocean vorticity at 20 meters depth normalized by the Coriolis parameter. The ice-free ocean is masked in black, and the land is masked in white. Note the sea ice transition from the viscous-plastic fluid regime that is accompanied by linear kinematic features towards a nearly passive tracer regime within and near marginal ice zones, particularly evident at the end of summer. This passive tracer regime spreads to occupy a significant portion of the Arctic sea ice. The high-resolution LLC4320 model is not a reanalysis-type model and hence was not adjusted to accurately reproduce observations. As a result, there is an apparent mismatch in the locations of summer sea ice retreat: compared to the observed 2012 sea ice state, the model overestimates the sea ice cover in the Western Arctic ocean and underestimates its melt in the Eastern Arctic. Nonetheless, the highresolution simulation provides a unique opportunity to explore the critical role of small-scale MIZ dynamics. These fundamental dynamical processes should be ubiquitous to the MIZ regardless of exact geographical location and size.
